# Neuroprotective Effects of Thymoquinone by the Modulation of ER Stress and Apoptotic Pathway in In Vitro Model of Excitotoxicity

**DOI:** 10.3390/molecules26061592

**Published:** 2021-03-13

**Authors:** Elisa Landucci, Costanza Mazzantini, Daniela Buonvicino, Domenico E. Pellegrini-Giampietro, Maria Camilla Bergonzi

**Affiliations:** 1 Department of Health Sciences, Section of Clinical Pharmacology and Oncology, University of Florence, Viale Pieraccini 6, 50139 Florence, Italy; costanza.mazzantini@unifi.it (C.M.); daniela.buonvicino@unifi.it (D.B.); domenico.pellegrini@unifi.it (D.E.P.-G.); 2 Department of Chemistry, University of Florence, Via Ugo Schiff 6, Sesto Fiorentino, 50019 Florence, Italy

**Keywords:** thymoquinone, excitotoxicity, neuroprotection, organotypic hippocampal slices, ER stress, apoptosis

## Abstract

Experimental evidence indicates that the activation of ionotropic glutamate receptors plays an important role in neurological disorders’ models such as epilepsy, cerebral ischemia and trauma. The glutamate receptor agonist kainic acid (KA) induces seizures and excitotoxic cell death in the CA3 region of the hippocampus. Thymoquinone (TQ) is the most important component of the essential oil obtained from black cumin (*Nigella sativa* L.) seeds. It has many pharmacological actions including antioxidant, anti-inflammatory, and anti-apoptotic effects. TQ was used in an in vitro experimental model of primary cultures where excitotoxicity was induced. Briefly, rat organotypic hippocampal slices were exposed to 5 µM KA for 24 h. Cell death in the CA3 subregions of slices was quantified by measuring propidium iodide fluorescence. The cross-talk between TQ, ER stress and apoptotic pathways was investigated by Western blot. In untreated slices TQ (10 µM) induced a significant increase on the PSD95 levels and it decreased the excitotoxic injury induced by KA. Additionally, TQ was able to ameliorate the KA-induced increase in unfolded proteins GRP78 and GRP94 expression. Finally, TQ was able to partially rescue the reduction of the KA-induced apoptotic pathway activation. Our results suggest that TQ modulates the processes leading to post-kainate neuronal death in the CA3 hippocampal area.

## 1. Introduction

In recent years, medicinal plants have been increasingly used in support or as an alternative to synthetic drugs and they have represented an important source of new compounds, due to the enormous chemical and structural diversity of their constituents. The reduced toxicity, availability and lower cost of medicinal plants and their derivatives when compared to many synthetic drugs, make them an interesting and sustainable alternative for the treatment and prevention of various diseases, including neurological ones. The neuroprotection of various plant compounds such as ginsenoside, baicalein, curcumin, resveratrol, peurarine and quercetin has been demonstrated in cellular or animal models. Thymoquinone (2-isopropyl-5-methylbenzo-1,4-quinone (TQ)) is the main constituent of the essential oil and one of the main compounds of the hydroalcoholic extract of *Nigella sativa* L. It was shown to have antioxidant [1,2], antimicrobial [3,4], immunomodulatory [5], antihistamine [6] and antinflammatory [7,8], hypoglycemic [9], hepatoprotective [10,11], antihypertensive [12], antidiabetic [13,14] and antioncogenic [15,16,17] properties. Numerous studies report the effectiveness of TQ in the treatment of neurological conditions such as epilepsy, Parkinson’s, Alzheimer’s, anxiety and learning and memory deficits [18]. Furthermore, through its antioxidant, anti-inflammatory [19] and anti-apoptotic effects [18], TQ protects brain cells from various injuries. In a recent review, Isaev and colleagues analysed the neuroprotective action of TQ in models of brain ischemia/reperfusion, Alzheimer’s and Parkinson’s diseases, and traumatic brain injury and concluded that is mediated by inhibition of lipid peroxidation, downregulation of proinflammatory cytokines, maintenance of mitochondrial membrane potential, and prevention of apoptosis through inhibition of caspases-3, -8, and -9 [20]. Moreover, TQ attenuates brain injury via anti-oxidative pathway in a status epilepticus rat model [21]. Numerous studies have reported that many natural compounds can induce neuronal differentiation, outgrowth, survival, and improved synaptic function in the central nervous system. Jang et al. have demonstrated that a mixture of *Schisandra chinensis* extract (SCE) and ascorbic acid (AA) improved cognitive function and induced synaptic plasticity-regulating proteins by enhancing mitochondrial respiration [22].

In particular, they have observed that injection of the SCE-AA mixture in mice significantly increased expression of postsynaptic density protein 95 (PSD95), an increase that was correlated with enhanced brain-derived neurotrophic factor (BDNF) expression. A recent study indicates a new correlation between ER stress, TRIB3 and AKT signaling and acute and prolonged hippocampal injury following KA-induced seizure, suggesting that the ER stress-associated gene TRIB3 plays an important role in the neuronal apoptosis triggered by seizures [23]. The protective effect of TQ was also studied in the context of the warm ischemia-reperfusion (I/R) injury model in the liver and it was observed that TQ helped preventing histological damages, inflammation, oxidative stress and contributed to the decreased expression of ER stress parameters [24].

The aim of our study was to evaluate neuroprotective activities of TQ in a pathological brain model of temporal lobe epilepsy (TLE), a type of epilepsy that involves neuronal loss, reactive astrogliosis and increased oxidative stress. For this reason, rat organotypic hippocampal slices were exposed to KA and the neuroprotective effects of TQ as well as the underlying mechanisms were evaluated. For this purpose, we performed a comprehensive analysis of the expression and phosphorylation levels of key plasticity and apoptotic regulators as synaptophysin and PSD95, the unfolded protein response (UPR)-related genes, eIF2α, BAX and PARP. Of note, our study outlines for the first time that TQ is able to induce an increase in PSD95 levels and to modulate the ER stress pathway in the context of seizures.

## 2. Results

### 2.1. Effect of TQ on PSD95 Levels

**Figure 1:**
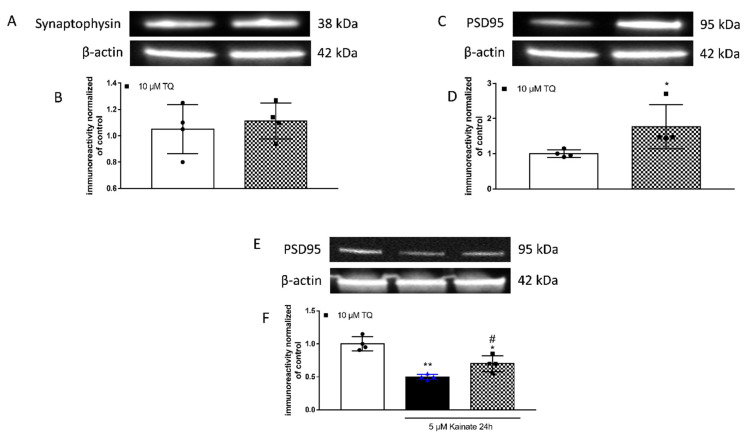
Effects of TQ on synaptophysin and PSD95 in organotypic hippocampal slice cultures in basal condition. Illustrative blots using antibodies directed against synaptophysin (**A**) and PSD95 (**C**,**E**) and β-actin. (B-D-F). Quantitative analysis of immunoreactive bands shows no significant changes in levels of synaptophysin (**B**), but a significant increase in PSD95 (**D**) induced by application of 10 µM TQ for 24 h in basal condition. TQ is able to prevent the decrease in PSD95 induced by kainate. Dot blots show the results of four experiments from independent slice preparations and each dot is the pool of four slices. * *p* < 0.05 vs. CRL. (Unpaired *t*-test in (**B**,**D**)) and * *p* < 0.05, ** *p* < 0.01 vs. CRL, # *p* < 0.05 vs. kainate. (ANOVA + Tukey’s *w*-Test in (**F**)).

To analyse whether TQ affected synaptic plasticity, hippocampal slices were treated with TQ at the concentration of 10 µM for 24 h in basal condition and the total protein were extracted and analysed using the Western blot technique. Compared to control untreated slices, we observed a significant increase in the expression of PSD95 induced by TQ treatment (Figure 1C,D), whereas no significant change was detected in the levels of synaptophysin, suggesting that TQ selectively modulates the expression of postsynaptic proteins while does not affect the presynaptic compartment (Figure 1A,B).

To investigate the effects of TQ in an in vitro model of epilepsy, we treated rat organotypic hippocampal slices with 5 µM KA for 24 h. This treatment induced a significant decrease of PSD95 protein levels that was significantly rectified by application of TQ (Figure 1E,F).

### 2.2. Effect of TQ on KA-Induced Neuronal Cell Death

**Figure 2:**
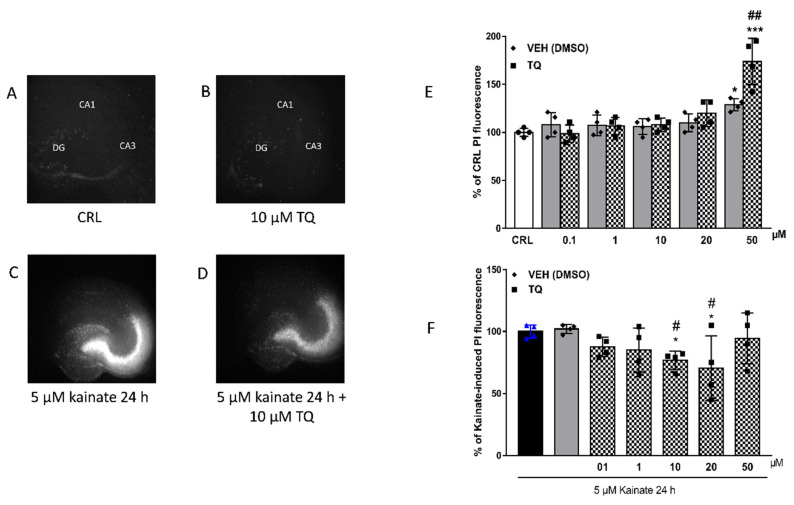
Effects of TQ in organotypic hippocampal slice cultures exposed to KA. (**A**) representative hippocampal slice under normal conditions (background PI fluorescence); representative slice incubated with 10 µM of TQ in basal condition (**B)**; representative slice exposed to 5 µM kainate 24 h displaying intense PI labelling in the CA3 subregion (**C**); TQ effect on KA-induced PI labelling (**D**). (**E**): Quantitative analysis of PI fluorescence measured after 24 h of incubation with TQ (0.1–50 µM) and its corresponding vehicle in basal condition. * *p* < 0.05, *** *p* < 0.001 vs. CRL and ## vs. corresponding vehicle (ANOVA + Tukey’s *w*-Test). (**F**) Quantitative analysis of PI fluorescence measured after 24 h of incubation with TQ (0.1-50 µM) and its corresponding vehicle in the Kainate-induced injury model. Bars represent the mean ± SEM of at least four experiments run in quadruplicate, from independent slice preparations (about 16 slices for each experimental point). * *p* < 0.05 vs. KA; # *p* < 0.05 vs. VEH (ANOVA + Tukey’s *w*-Test).

To investigate the safety and tolerability of TQ, rat organotypic hippocampal slices were incubated with increasing concentrations of TQ and the Cornu Ammonis areas CA3 region was evaluated for damage using PI fluorescence (Figure 2A,B,E). TQ was solubilized in dimethyl sulfoxide at the concentration of 5 mg/mL, diluted in the medium and incubated during the 24 h of the treatment. Quantitative analysis of hippocampal slices exposed to TQ (0.1–50 µM) for 24 h showed that the drug did not induce injury in the CA3 region in this model as compared to the vehicle-treated or the control group. Only the dose of 50 µM and its corresponding vehicle (DMSO 0.16%) resulted in toxicity as compared to the control (Figure 2E) (* *p* < 0.05 and *** *p* < 0.001 vs. CRL and ## vs. corresponding vehicle). These results showed that TQ was well-tolerated.

The effects of TQ in organotypic hippocampal slice cultures exposed to KA were also evaluated. As expected, KA-induced cell death in the CA3 region of the hippocampus (Figure 2C). The KA-induced toxic effect was reduced in slices pre-exposed to increasing concentrations of TQ, which became significantly neuroprotective at the concentration of 10 µM and 20 µM. While TQ treatment was no longer neuroprotective at 50 µM, we did not observe a synergic detrimental effect between KA and TQ 50 µM (* *p* < 0.05 vs. KA; # *p* < 0.05 vs. VEH Figure 2F) (Figure 2D,F).

**Figure 3:**
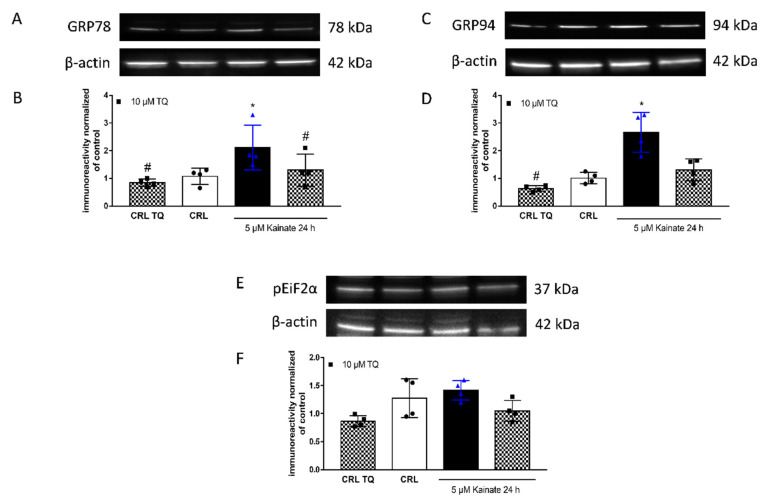
Effects of TQ on unfolded protein response (UPR)-related genes and phosphorylated eIF2α in slices. Slices were exposed to 10 µM of TQ in the absence or presence of KA (5 µM for 24 h) and then processed for WB. (**A**,**C**,**E**): Representative blots for GRP78 or GRP94 or pEiF2α or β-actin. (**B**,**D**,**F**): Quantitative analysis of immunoreactive bands shows that TQ treatment in basal conditions does not change the total proteins levels but is able to reduce KA-induced enhancement of GRP78 and GRP94 levels, without changing the phosphorylation state of eIF2α. Dot blots show the results of four experiments from independent slice preparations and each dot is the pool of four slices. * *p* < 0.05 vs. CRL and # *p* < 0.05 vs. kainate. (ANOVA + Tukey’s *w*-Test).

To investigate the intracellular regulators underlying the TQ neuroprotective effects, we looked at the expression levels and phosphorylation states of a few key regulators of synaptic plasticity and apoptic pathways. Interestingly we found that 24 h of TQ application in basal condition decreased, although not significantly, the level of unfolding protein response (UPR)-related genes. Similar to what was reported in a model of organotypic hippocampal slices exposed to oxygen glucose deprivation [25], KA application induced a significant increase in unfolded proteins, in particular GRP78 (Figure 3A,B) and GRP94 (Figure 3C,D). This effect was partly abolished in slices that were pre-exposed to TQ. Conversely, ANOVA statistical analysis of Western blot assays showed that phosphorylation of the eukaryotic translation initiation factor 2 subunit alpha (eIF2a) did not significantly change between the basal condition or kainite-induced toxic model, independently from TQ treatment (Figure 3E,F).

### 2.3. Effect of TQ on KA-Induced Apoptosis

The role of TQ on apoptosis in the nervous system is known. Ullah and colleagues studied the effects of TQ and vitamin C against PTZ-induced generalized seizures in rats and observed that TQ decreases Bcl-2 expression and activates caspase-3 in the cortex and in the hippocampus [26].

**Figure 4:**
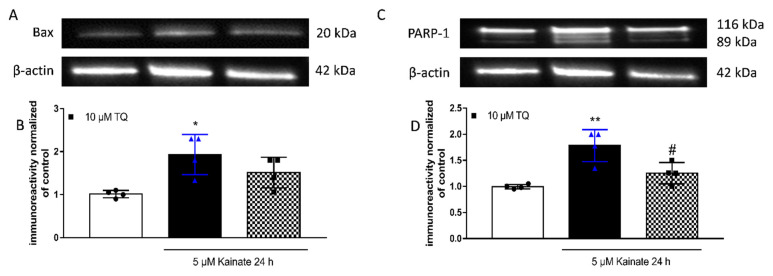
Effects of TQ on Bax and PARP-1 in slices exposed to KA. Slices were exposed to the KA (5 µM for 24 h) and then processed for WB. TQ was added to the incubation medium during KA exposure. (**A**,**C**): Representative blots for Bax or PARP-1 or β-actin. (**B**,**D**): Quantitative analysis of immunoreactive bands, showing that TQ is able to reduce KA-induced enhancement in PARP-1 expression levels while having no effects on Bax. Dot blots show the results of four experiments from independent slice preparations and each dot is the pool of four slices. * *p* < 0.05 and ** *p* < 0.01 vs. CRL, # *p* < 0.05 vs. kainate. (ANOVA + Tukey’s *w*-Test).

We evaluated whether TQ had any anti-apoptotic effect by looking at the expression levels of BAX and of cleaved PARP using our KA-induced toxicity model; in parallel with previous studies, we report that TQ has an anti-apoptotic effect in rat organotypic hippocampal slices (Figure 4).

## 3. Discussion

KA triggers a delayed type of excitotoxic cell death in the CA3 area of organotypic hippocampal slices through activation of ionotropic glutamate receptors [27,28]. Additionally, it has been shown that KA induces cell death through caspases activation [29], increase expression of pro-apoptotic proteins, such as Bim and Bax, which lead to a decrease in the Bcl-2-Bad ratio, paralleled release of cytochrome c and cleavage of poly(ADP-ribose)-polymerase (PARP) [30]. Current antiepileptic drugs have limited efficacy and provide little or no benefits in 30% of the patients; the potential of anti-inflammatory drugs should be explored in order to provide additional therapeutic approaches that can help preventing or reducing epileptogenesis [31]. Preclinical (both in vivo and in vitro) and clinical epilepsy studies we conducted, in which nonsteroidal anti-inflammatory drugs (NSAIDs), such as cyclooxygenase-2 (COX-2) selective inhibitors (COXIBs) and nonselective NSAIDs were used to control seizure. Different natural compounds have been shown to display anti-oxidative, anti-inflammatory, and anti-apoptotic activities and they are recommended as a supplementation for prevention and/or treatment of neurological disorders; among these compounds we can find natural molecules such as anthocyanins [23], curcumin [32] eugenol and naringin [33] and TQ [20]. TQ has promising effects against a variety of inflammatory diseases and cancer. TQ modulates several major signaling pathways (such as NF-kB, STATs and Wnt/β-catenin) and key oncogenic molecules that play a prominent role in cancer [34]. Various studies have reported that TQ can enhance the efficacy of chemotherapeutic agents while reducing their toxic side effects. In addition, TQ inhibits the growth of breast, prostate, pancreatic, colon, lung, and hematological malignancies in different mouse models of cancer [35]. In accordance with our data, Firdaus et colleagues revealed that TQ (10 and 20 μM) could prevent neurotoxicity arsenic-mediated apoptosis and cytotoxicity, by either decreasing or increasing the levels of Bax or Bcl-2 respectively [36]. Moreover, TQ has been shown to have a protective activity via MAP kinase signaling pathways against neurotoxicity mediated by Acrylamide in rats’ cortex [37,38]. In this study, for the first time, we show the neuroprotective properties of TQ in a pathological brain model of temporal lobe epilepsy (TLE) using organotypic hippocampal slices exposed to KA. We found that TQ induced an increase of the key plasticity protein PSD95 in basal condition and that it was able to regulate ER stress signalling pathway in the seizure model (KA).

Our findings evidenced that 24 h treatment with 10 µM of TQ induces a significant increase in the level of PSD95 in organotypic hippocampal slices, suggesting increased synaptic activity. In fact, it is known that PSD95 has been implicated in synaptic development, plasticity [39], and a decrease of its levels is associated with several brain disorders [40]. By regulating the organization of receptors and related proteins, PSD95 is involved in maturation of cortical circuits, advanced brain functions such as learning and memory—all of which presumably reflect the crucial roles of PSD95 in synaptic plasticity [41]. It has been shown that status epilepticus alters the expression of synaptic proteins that may enhance presynaptic neurotransmitter release and postsynaptic receptor sensitivity [42]. Downregulation of the PSD95 was reported after epileptic seizures, associated with neuronal loss in the entire hippocampus [41,43]. Sun and colleagues suggested that down-regulation of PSD95 in the hippocampus in rats with long-term spontaneous recurrent seizure was correlated with the observed behavioural deficits, including spatial learning memory deficit, anxiety and increased locomotor activity [44].

The neuronal degeneration mediated by KA involves ER stress and the activation of ER sensors [45]. In organotypic hippocampal slices, KA induces a selective death in the CA3 region [27,28,46]. We observed that at a concentration of 10 or 20 µM, TQ was able to reduce neuronal damage induced by KA (Figure 2F). The neuroprotective effect is correlated with its ability to reduce the KA-induced increased expression of chaperon unfolding proteins GRP78 and GRP94 (Figure 3). Previously, Sokka showed that Salubrinal leads to an inhibition of ER stress and this results in neuroprotective effects in vivo [45]. The UPR reprograms gene expression in order to regulate proteostasis or induce apoptosis of irreversibly damaged cells [47]. When the capacity of the UPR to sustain proteostasis is overwhelmed, cells enter apoptotic programmes [48]. Numerous mechanisms have been proposed to sensitize cells to ER stress-induced apoptosis, where a network of upstream events rather than a single pathway controls cell demise under irreversible ER damage [49]. Faced within the linear interaction between ER stress and apoptosis, we analysed by western blot the level of Bax and PARP1 as key apoptosis signaling molecules and we observed that TQ dampens the activation of the apoptosis pathway by reversing the increase in PARP-1 expression levels induced by KA.

## 4. Materials and Methods

### 4.1. Animals

Male and female Wistar rat pups (7–9 days old) were obtained from Charles River (MI, Italy). Animals were housed at 23 ± 1 °C under a 12 h light-dark cycle (lights on at 07:00) and were fed a standard laboratory diet with ad libitum access to water. Experiments and animal use procedures were carried out in accordance with the National Institutes of Health Guide for the Care and Use of Laboratory Animals (NIH Publications No. 80-23, revised 1996). The authors further attest that all efforts were made to minimize the number of animals used and their suffering. We used 24 pups for Western blot analysis and 36 pups for experiments of neurotoxic-neuroprotective activity.

### 4.2. Materials

Propidium iodide (PI) and TQ were purchased from Sigma-Aldrich (ST: Louis, MO, USA). Tissue culture reagents were obtained from Gibco-BRL (San Giuliano Milanese, Milan, Italy) and Sigma (St. Louis, MO, USA).

### 4.3. Preparation of Rat Organotypic Hippocampal Slice Cultures

Organotypic hippocampal slice cultures were prepared as previously reported [50,51]. Briefly, hippocampi were removed from the brains of 7–9 days old Wistar rats (Charles River, MI, Italy), and transverse slices (420 μm) were prepared using a McIlwain tissue chopper and transferred onto semiporous membranes inserts and maintained in culture for 14 days in vitro. We usually obtain about eight slices for each pup. Slices were exposed to vehicle or 10 µM TQ for 24 h. The slices were incubated with 5 µM KA [27,28,46]. TQ was solubilized in dimethyl sulfoxide at the concentration of 5 mg/mL, diluted in the medium and incubated during the 24 h of the treatment. Cell death was evaluated by using the fluorescent dye propidium iodide (5 μg/mL) in live tissue and fluorescence was viewed using an inverted fluorescence microscope. Images were analysed using morphometric analysis software. Cellular death, the CA3 hippocampal subfields were identified respectively for KA toxicity has been quantified by the image software (ImageJ; NIH, Bethesda, MD, USA) which had detected the optical density of PI fluorescence (the fluorescence measured in KA-exposed slices in the CA3 region was reported as 100%).

### 4.4. Western Blot Analysis

Western blotting was conducted as previously reported [25]. Four slices for the sample were dissolved in 1% SDS. BCA (bicinchoninic acid) Protein Assay were used to quantify the total protein levels. Lysates (20 μg/lane of protein) were resolved by electrophoresis on a 4–20% SDS-polyacrylamide gel (Bio-Rad Laboratories, Hercules, CA, USA) and transferred onto nitrocellulose membranes. After blocking, the blots were incubated overnight at 4 °C with GRP94 and pEIF2α (from Abcam, Cambridge, London, UK), PARP-1, BAX and PSD95 (from Cell Signaling Technology, Beverly, MA, USA), GRP78 (Invitrogen, Carlsbad, CA, USA) and monoclonal-mouse antibody against Synaptophysin (Sigma-Merk, Darmstadt, Germany) diluted 1:1000 in TBS-T containing 5% bovine serum albumin. Β-actin was used as a loading control (monoclonal antibody purchased from Sigma (St. Louis, MO, USA). Immunodetection was performed with HRP-conjugated secondary antibodies (1:2000 anti-mouse, anti-rabbit or anti-goat IgG from donkey (Amersham Biosciences, Little Chalfont, UK) in TBS-T containing 5% non-fat dry milk. After washing, the membranes and reactive bands were detected using chemiluminescence (ECLplus; Euroclone, Padova, Italy). Quantity One analysis software was used for quantitative analysis (Bio-Rad, Hercules, CA, USA). Results are presented as the mean standard error of the mean (SEM) of different gels and expressed as AU-, which depicts the ratio between levels of target protein expression and β-actin normalized to basal levels.

### 4.5. Statistical Analysis

Data are presented as means ± SEM of n experiments. The statistical significance of differences between PI fluorescence intensity or Western blot optical densities was analysed using Unpaired t-test or one-way ANOVA with a post hoc Tukey’s *w*-test for multiple comparisons. All statistical calculations were performed using GraphPad Prism v. 7 for Windows (GraphPad Software, San Diego, CA, USA). A probability value (*p*) of <0.05 was considered significant.

## 5. Conclusions

TQ has a safety profile and is well tolerated for the treatment of neurological diseases. Many preclinical studies using in vitro and in vivo models of neurological damage show that TQ is an efficient and promising novel and natural neuroprotective agent. In this study, the neuroprotective effect of TQ was evaluated in pathological brain model of temporal lobe epilepsy, which is a type of epilepsy that involves neuronal loss. TQ increases the levels of PSD95 in basal conditions. These findings support the idea that the TQ, alone or in combination with classical antiepileptic drugs, may become a valid and safe therapeutic intervention in the treatment of neurological disorders.

## Data Availability

The data presented in this study are available on request from the corresponding author.

## Sample Availability

Samples of the compounds are available from the authors.

## Abbreviations

TQ, thymoquinone;
SCE, *Schisandra chinensis extract*;
AA, ascorbic acid;
PSD95, postsynaptic density protein 95;
BDNF, brain-derived neurotrophic factor;
ER stress, Endoplasmic Reticulum stress;
TRIB3, tribblespseudokinase 3;
AKT, protein kinase B;
KA, kainic acid;
I/R, ischemia-reperfusion;
TLE, temporal lobe epilepsy;
CA3, Cornu Ammonis 3;
WB, Western blotting;
GRP78, Glucose Regulated Protein 78;
GRP94, Glucose Regulated Protein 94;
eIF2a, eukaryotic translation initiation factor 2 subunit alpha;
PTZ, pentylenetetrazole;
Bcl-2, B-cell lymphoma 2;
BAX, Bcl-2 Associated X-protein;
PARP, Poly [ADP-ribose] polymerase;
PARP-1, Poly [ADP-ribose]polymerase 1;
Bim, Bcl-2-like protein 11;
Bad, BCL2 associated agonist of cell death;
NSAIDs, nonsteroidal anti-inflammatory drugs;
COX-2, cyclooxygenase-2;
COXIBs, cyclooxygenase-2 selective inhibitors;
UPR, unfolded protein response;
PI, Propidium iodide;
SDS, Sodium Dodecyl Sulphate;
BCA, bicinchoninic acid;
TBS-T, Tris Buffered Saline-Tween;
HRP, Horseradish Peroxidase;
ECL-plus, Enhanced ChemiLuminescence;
AU, arbitrary units;
SEM, standard error of the mean.

## References

[1] Mansour M.A., Nagi M.N., El-Khatib A.S., Al-Bekairi A.M. (2002). Effects of thymoquinone on antioxidant enzyme activities, lipid peroxidation and dt-diaphorase in different tissues of mice: A possible mechanism of action. Cell Biochem. Funct..

[2] Badary O.A., Taha R.A., El-Din A.M.G., Abdel-Wahab M.H. (2003). Thymoquinone is a potent superoxide anion scavenger. Drug Chem. Toxicol..

[3] Chaieb K., Kouidhi B., Jrah H., Mahdouani K., Bakhrouf A. (2011). Antibacterial activity of Thymoquinone, an active principle of Nigella sativa and its potency to prevent bacterial biofilm formation. BMC Complement. Altern. Med..

[4] Kouidhi B., Zmantar T., Jrah H., Souiden Y., Chaieb K., Mahdouani K., Bakhrouf A. (2011). Antibacterial and resistance-modifying activities of thymoquinone against oral pathogens. Ann. Clin. Microbiol. Antimicrob..

[5] Salem M.L. (2005). Immunomodulatory and therapeutic properties of the Nigella sativa L. seed. Int. Immunopharmacol..

[6] Mahgoub A.A. (2003). Thymoquinone protects against experimental colitis in rats. Toxicol. Lett..

[7] Houghton P.J., Zarka R., De Las Heras B., Hoult J.R.S. (1995). Fixed oil of Nigella sativa and derived thymoquinone inhibit eicosanoid generation in leukocytes and membrane lipid peroxidation. Planta Med..

[8] El Gazzar M., El Mezayen R., Marecki J.C., Nicolls M.R., Canastar A., Dreskin S.C. (2006). Anti-inflammatory effect of thymoquinone in a mouse model of allergic lung inflammation. Int. Immunopharmacol..

[9] El-Mahmoudy A., Shimizu Y., Shiina T., Matsuyama H., El-Sayed M., Takewaki T. (2005). Successful abrogation by thymoquinone against induction of diabetes mellitus with streptozotocin via nitric oxide inhibitory mechanism. Int. Immunopharmacol..

[10] Nagi M.N., Almakki H.A. (2009). Thymoquinone supplementation induces quinone reductase and glutathione transferase in mice liver: Possible role in protection against chemical carcinogenesis and toxicity. Phyther. Res..

[11] Alenzi F.Q., El-Sayed El-Bolkiny Y., Salem M.L. (2010). Protective effects of Nigella sativa oil and thymoquinone against toxicity induced by the anticancer drug cyclophosphamide. Br. J. Biomed. Sci..

[12] Khattab M.M., Nagi M.N. (2007). Thymoquinone supplementation attenuates hypertension and renal damage in nitric oxide deficient hypertensive rats. Phyther. Res..

[13] Pari L., Sankaranarayanan C. (2009). Beneficial effects of thymoquinone on hepatic key enzymes in streptozotocin-nicotinamide induced diabetic rats. Life Sci..

[14] Hawsawi Z.A., Ali B.A., Bamosa A.O. (2001). Effect of Nigella sativa (black seed) and thymoquinone on blood glucose in albino rats. Ann. Saudi Med..

[15] Banerjee S., Padhye S., Azmi A., Wang Z., Philip P.A., Kucuk O., Sarkar F.H., Mohammad R.M. (2010). Review on molecular and therapeutic potential of thymoquinone in cancer. Nutr. Cancer.

[16] Shoieb A.M., Elgayyar M., Dudrick P.S., Bell J.L., Tithof P.K. (2003). In vitro inhibition of growth and induction of apoptosis in cancer cell lines by thymoquinone. Int. J. Oncol..

[17] Woo C.C., Loo S.Y., Gee V., Yap C.W., Sethi G., Kumar A.P., Tan K.H.B. (2011). Anticancer activity of thymoquinone in breast cancer cells: Possible involvement of PPAR-γ pathway. Biochem. Pharmacol..

[18] Samarghandian S., Farkhondeh T., Samini F. (2018). A Review on Possible Therapeutic Effect of Nigella sativa and Thymoquinone in Neurodegenerative Diseases. CNS Neurol. Disord. Drug Targets.

[19] Ragheb A., Attia A., Eldin W.S., Elbarbry F., Gazarin S., Shoker A. (2009). The protective effect of thymoquinone, an antioxidant and anti-inflammatory agent, against renal injury: A review. Saudi J. Kidney Dis. Transpl..

[20] Isaev N.K., Chetverikov N.S., Stelmashook E.V., Genrikhs E.E., Khaspekov L.G., Illarioshkin S.N. (2020). Thymoquinone as a Potential Neuroprotector in Acute and Chronic Forms of Cerebral Pathology. Biochemistry.

[21] Shao Y.Y., Li B., Huang Y.M., Luo Q., Xie Y.M., Chen Y.H. (2017). Thymoquinone attenuates brain injury via an antioxidative pathway in a status epilepticus rat model. Transl. Neurosci..

[22] Jang Y., Lee J.H., Lee M.J., Kim S.J., Ju X., Cui J., Zhu J., Lee Y.L., Namgung E., Sung H.W.J. (2020). Schisandra extract and ascorbic acid synergistically enhance cognition in mice through modulation of mitochondrial respiration. Nutrients.

[23] Zhang J., Wu J., Liu F., Tong L., Chen Z., Chen J., He H., Xu R., Ma Y., Huang C. (2019). Neuroprotective effects of anthocyanins and its major component cyanidin-3-O-glucoside (C3G) in the central nervous system: An outlined review. Eur. J. Pharmacol..

[24] Bouhlel A., Mosbah B., Abdallah H., Ribault C., Viel R., Mannaï S., Corlu A., Abdennebi B. (2017). Thymoquinone prevents endoplasmic reticulum stress and mitochondria-induced apoptosis in a rat model of partial hepatic warm ischemia reperfusion. Biomed. Pharmacother..

[25] Landucci E., Llorente I.L., Anuncibay-Soto B., Pellegrini-Giampietro D.E., Fernández-López A. (2018). Bicuculline Reverts the Neuroprotective Effects of Meloxicam in an Oxygen and Glucose Deprivation (OGD) Model of Organotypic Hippocampal Slice Cultures. Neuroscience.

[26] Ullah I., Badshah H., Naseer M.I., Lee H.Y., Kim M.O. (2015). Thymoquinone and Vitamin C Attenuates Pentylenetetrazole-Induced Seizures Via Activation of GABAB1 Receptor in Adult Rats Cortex and Hippocampus. Neuromol. Med..

[27] Landucci E., Gencarelli M., Mazzantini C., Laurino A., Pellegrini-Giampietro D.E., Raimondi L. (2019). N-(3-Ethoxy-phenyl)-4-pyrrolidin-1-yl-3-trifluoromethyl-benzamide (EPPTB) prevents 3-iodothyronamine (T1AM)-induced neuroprotection against kainic acid toxicity. Neurochem. Int..

[28] Landucci E., Pellegrini-Giampietro D.E., Bilia A.R., Bergonzi M.C. (2019). Enhanced neuroprotective effects of panax ginseng g115^®^ and ginkgo biloba gk501^®^ combinations in vitro models of excitotoxicity. Int. J. Mol. Sci..

[29] Korhonen L., Belluardo N., Lindholm D. (2001). Regulation of X-chromosome-linked inhibitor of apoptosis protein in kainic acid-induced death in the rat hippocampus. Mol. Cell. Neurosci..

[30] Zhao Y., Spigolon G., Bonny C., Culman J., Vercelli A., Herdegen T. (2012). The JNK inhibitor D-JNKI-1 blocks apoptotic JNK signaling in brain mitochondria. Mol. Cell. Neurosci..

[31] Radu B.M., Epureanu F.B., Radu M., Fabene P.F., Bertini G. (2017). Nonsteroidal anti-inflammatory drugs in clinical and experimental epilepsy. Epilepsy Res..

[32] Dhir A. (2018). Curcumin in epilepsy disorders. Phyther. Res..

[33] Kim S.R. (2016). Control of Granule Cell Dispersion by Natural Materials Such as Eugenol and Naringin: A Potential Therapeutic Strategy Against Temporal Lobe Epilepsy. J. Med. Food.

[34] Shanmugam M.K., Arfuso F., Kumar A.P., Wang L., Goh B.C., Ahn K.S., Bishayee A., Sethi G. (2018). Modulation of diverse oncogenic transcription factors by thymoquinone, an essential oil compound isolated from the seeds of Nigella sativa Linn. Pharmacol. Res..

[35] Shanmugam M.K., Ahn K.S., Hsu A., Woo C.C., Yuan Y., Tan K.H.B., Chinnathambi A., Alahmadi T.A., Alharbi S.A., Koh A.P.F. (2018). Thymoquinone Inhibits Bone Metastasis of Breast Cancer Cells Through Abrogation of the CXCR4 Signaling Axis. Front. Pharmacol..

[36] Firdaus F., Zafeer M.F., Anis E., Ahmad F., Hossain M.M., Ali A., Afzal M. (2019). Evaluation of phytomedicinal efficacy of thymoquinone against Arsenic induced mitochondrial dysfunction and cytotoxicity in SH-SY5Y cells. Phytomedicine.

[37] Tabeshpour J., Mehri S., Abnous K., Hosseinzadeh H. (2019). Neuroprotective Effects of Thymoquinone in Acrylamide-Induced Peripheral Nervous System Toxicity Through MAPKinase and Apoptosis Pathways in Rat. Neurochem. Res..

[38] Tabeshpour J., Mehri S., Abnous K., Hosseinzadeh H. (2020). Role of Oxidative Stress, MAPKinase and Apoptosis Pathways in the Protective Effects of Thymoquinone Against Acrylamide-Induced Central Nervous System Toxicity in Rat. Neurochem. Res..

[39] Zhang P., Lisman J.E. (2012). Activity-dependent regulation of synaptic strength by PSD-95 in CA1 neurons. J. Neurophysiol..

[40] De Bartolomeis A., Latte G., Tomasetti C., Iasevoli F. (2014). Glutamatergic postsynaptic density protein dysfunctions in synaptic plasticity and dendritic spines morphology: Relevance to schizophrenia and other behavioral disorders pathophysiology, and implications for novel therapeutic approaches. Mol. Neurobiol..

[41] Jiang Q., Wang J., Wu X., Jiang Y. (2007). Alterations of NR2B and PSD-95 expression after early-life epileptiform discharges in developing neurons. Int. J. Dev. Neurosci..

[42] Morimoto K., Fahnestock M., Racine R.J. (2004). Kindling and status epilepticus models of epilepsy: Rewiring the brain. Prog. Neurobiol..

[43] Frasca A., Aalbers M., Frigerio F., Fiordaliso F., Salio M., Gobbi M., Cagnotto A., Gardoni F., Battaglia G.S., Hoogland G. (2011). Misplaced NMDA receptors in epileptogenesis contribute to excitotoxicity. Neurobiol. Dis..

[44] Sun Q.J., Duan R.S., Wang A.H., Shang W., Zhang T., Zhang X.Q., Chi Z.F. (2009). Alterations of NR2B and PSD-95 expression in hippocampus of kainic acid-exposed rats with behavioural deficits. Behav. Brain Res..

[45] Sokka A.L., Putkonen N., Mudo G., Pryazhnikov E., Reijonen S., Khiroug L., Belluardo N., Lindholm D., Korhonen L. (2007). Endoplasmic reticulum stress inhibition protects against excitotoxic neuronal injury in the rat brain. J. Neurosci..

[46] Laurino A., Landucci E., Resta F., De Siena G., Pellegrini-Giampietro D.E., Masi A., Mannaioni G., Raimondi L. (2018). Anticonvulsant and Neuroprotective Effects of the Thyroid Hormone Metabolite 3-Iodothyroacetic Acid. Thyroid.

[47] Wang M., Kaufman R.J. (2016). Protein misfolding in the endoplasmic reticulum as a conduit to human disease. Nature.

[48] Ron D., Walter P. (2007). Signal integration in the endoplasmic reticulum unfolded protein response. Nat. Rev. Mol. Cell Biol..

[49] Hetz C., Zhang K., Kaufman R.J. (2020). Mechanisms, regulation and functions of the unfolded protein response. Nat. Rev. Mol. Cell Biol..

[50] Gerace E., Landucci E., Scartabelli T., Moroni F., Pellegrini-Giampietro D.E. (2012). Rat hippocampal slice culture models for the evaluation of neuroprotective agents. Methods Mol. Biol..

[51] Landucci E., Filippi L., Gerace E., Catarzi S., Guerrini R., Pellegrini-Giampietro D.E. (2018). Neuroprotective effects of topiramate and memantine in combination with hypothermia in hypoxic-ischemic brain injury in vitro and in vivo. Neurosci. Lett..

